# Preoperative Super-Selective Embolization of Carotid Body Tumor and Multidisciplinary Approach

**DOI:** 10.7759/cureus.12879

**Published:** 2021-01-23

**Authors:** Mohamed Selim, Shahad H AlJehani, Alaa B Aljuhani, Amr Awad Albayomy

**Affiliations:** 1 Vascular Surgery, Al Azhar Faculty of Medicine, Cairo, EGY; 2 Vascular Surgery, King Fahad University Hospital, Dammam, SAU; 3 Surgery, Imam Abdulrahman Bin Faisal University, Dammam, SAU; 4 Vascular Surgery, King Fahad Hospital of University, Khobar, SAU

**Keywords:** carotid body tumor, multidisciplinary approach, selective embolization, preoperative embolization

## Abstract

A carotid body tumor (CBT) is a rare highly vascular tumor. Therefore, the assessment of the tumor’s vessels preoperatively is significant to decrease the risk of vascular complications intraoperatively. We report a case of carotid body tumor in a 40-year-old female who presented with a headache and a pulsatile right neck mass. She underwent computed tomography (CT), which demonstrated a well-circumscribed tumor mass measuring 3 X 2.7 X 3.6 cm. Preoperatively, selective embolization was performed in which Onyx 18 (Medtronic, Dublin, Ireland) and precipitating hydrophobic injectable liquid (PHIL) 25% (Microvention Inc, California) were used, and that achieved 95% embolization of tumor blood supply. One day after embolization, complete surgical resection was done by a multidisciplinary team.

## Introduction

A carotid body tumor (CBT) is a rare neuroendocrine neoplasm, arising from the chemoreceptor cells of the carotid bulb. Around 35% of carotid body tumors are hereditary; malignant tumors account for 5%-7% of CBTs and the greatest risk is in young patients with familial tumors. An asymptomatic lateral neck mass is the usual presentation of CBTs, commonly in patients between the fifth and seventh decades. Other complaints can be nonspecific such as a sense of tightness in the neck, stiffness, or neck tenderness; rarely, patients present with symptoms related to endocrine hormone release such as palpitation, headache, diaphoresis, dizziness, and flushing [1-2].

With time, the size of CBTs may increase, leading to the invasion of the adjacent neurovascular structure. Consequently, the morbidity and complications of surgical resection rise. Therefore, the Shamblin classification has been developed to predict the difficulties and complications for surgical resection by describing the tumor in relation to the blood vessels. There are three groups: I, the tumor is less than 5 cm and relatively free of vessel involvement; II, the tumor is intimately involved but does not encase the vessel wall; and III, the tumor is intramural and encases the carotid vessels and adjacent nerves.

Imaging studies are important to confirm the diagnosis and delineate the relationship to the adjacent structure. However, localization of the tumor to the carotid bifurcation can be done by carotid duplex scan [3].

Assessment of the vessel's invasion and intracranial circulation using arteriography gives the benefit for preoperative embolization of the feeder vessels, which has shown to reduce intraoperative blood loss, better tumor visualization, and tumor shrinkage. In theory, preoperative embolization decreases the risk of stroke and neurological morbidity [3-5].

The recommended treatment of CBTs is surgical resection, and the importance of a multidisciplinary team approach has been reported in a cohort study that showed less morbidity and neurovascular complications despite the fact that included cases were with more advanced tumors [6].

In this paper, a case of carotid body tumor is reported, which required a multidisciplinary team to perform super-selective embolization followed by surgical resection.

## Case presentation

A 40-year-old woman was admitted under neurosurgery in King Fahad University Hospital with complaints of headache and a pulsatile right neck mass for eight years. Her symptoms were associated with constant right ear pain radiating to the jaw for six months duration. She denied experiencing compressive symptoms (dysphagia, dyspnea, or change in voice) and there was no history of aspiration or limited neck motion. On physical examination, a pulsating 2x2 cm mass was observed on the right side of the neck at level II, with no limitation of neck movement. Flexible nasopharyngoscopy was performed, which revealed a clear nasopharynx, oropharynx, mobile vocal folds, and no secretion in the supraglottic area. Cranial nerves examination revealed no abnormal findings.

Investigations at admission were normal, including CBC, renal function tests (RFTs), and liver function tests (LFTs).

**Figure 1:**
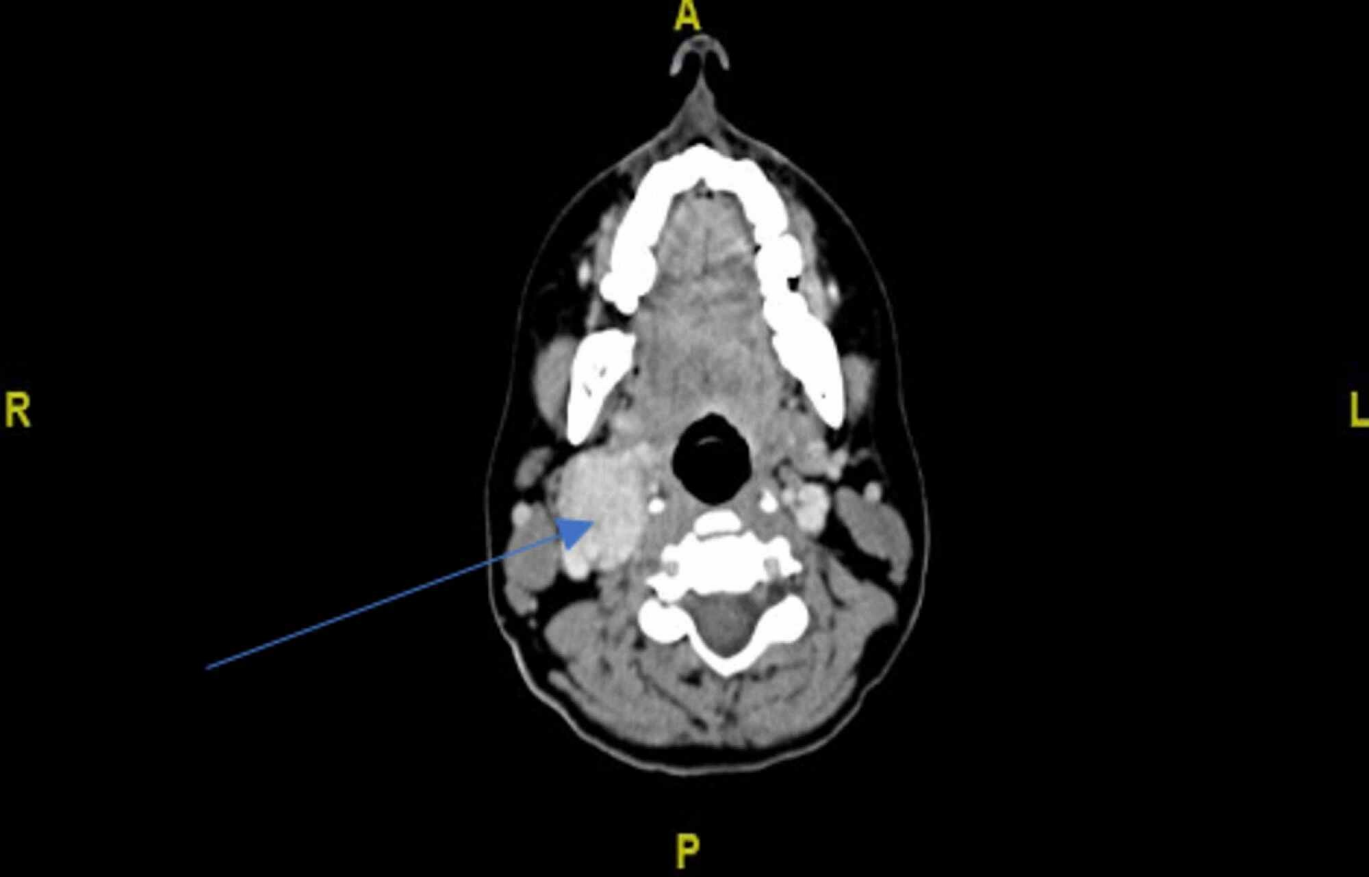
Contrast-enhanced computed tomography demonstrated a focal, rounded soft tissue mass of size 3 X 2.7 X 3.6 cm and located along the bifurcation of the right common carotid artery with splaying of external and internal carotid artery

She underwent computed tomography (CT) scan of the neck with and without contrast, which showed a focal, rounded soft tissue mass measuring 3 X 2.7 X 3.6 cm. It was located along the right common carotid artery (CCA) bifurcation with splaying of the internal carotid artery (ICA) and external carotid artery (ECA), which was consistent with a ShamblinII Carotid body tumor (Figure 1).

Pre-resection angiogram combined with selective embolization of the feeders was performed under aseptic technique and fluoroscopic guidance, in which Onyx 18 (Medtronic, Dublin, Ireland) and precipitating hydrophobic injectable liquid (PHIL) 25% (Microvention Inc, California) were used.

**Figure 2:**
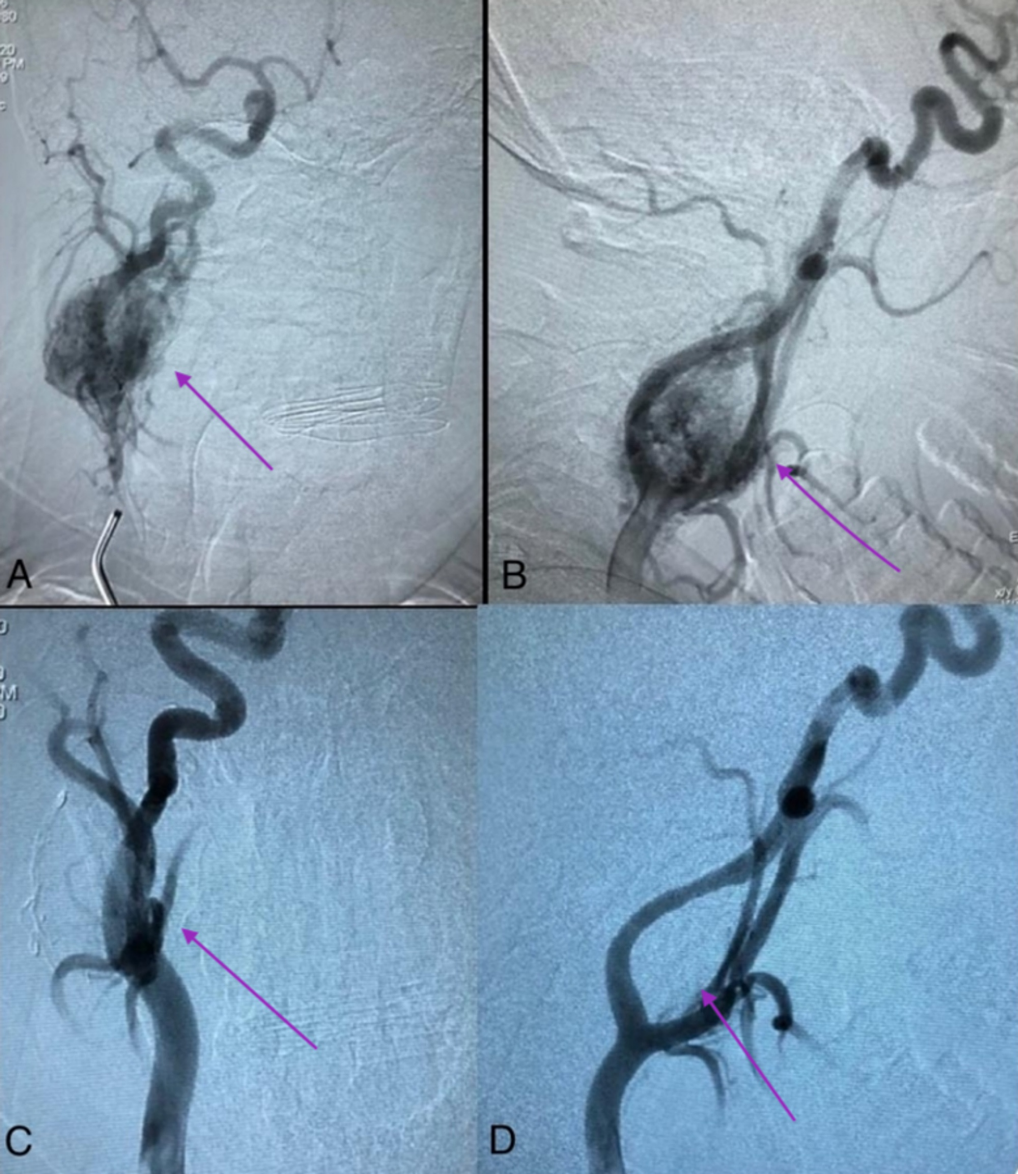
A, B: Preoperative angiography shows a vascularized CBT; C, D: Postembolization achieved devascularization of the tumor’s blood supply CBT: carotid body tumor

 A 5F sheath was inserted into the right femoral artery. A 5F vertebral catheter was used for selective catheterization of intracranial vessels. The 5F vertebral catheter was parked at the CCA proximal to the origin of the ECA. Selective runs and super-selective micro-runs were obtained to identify the optimal feeders to embolize. Using the specter C balloon microcatheter positioned at the distal occipital branch, micro-runs were taken before and during balloon inflation and while the specter balloon was inflated, Onyx 18 was injected into the feeders until reflex was observed. Then, an Echelon 14 was advanced into the distal feeder of the ascending pharyngeal branch and PHIL 25% was injected. This was followed by an Echelon 10 and PHIL 25% was injected into the main feeders from the ascending pharyngeal branch. Finally, an Echelon 14 was maneuvered into the proximal branch of the ECA, just distal to the bifurcation, and it was identified as the main feeder and serial runs were obtained; the micro-catheter was advanced into the tumor blush under roadmap guidance (Figure 2).

Due to non-aspiration of back bleeding into the micro-catheter, it was retracted more proximally sequentially till the proximal end of the feeding artery, at which point a control run showed no flow in the distal feeder and non-visualization of the tumor blush most likely due to spasm. This was maintained at control runs of 15 minutes. Control runs showed embolization of 95% of the carotid body tumor supply with few direct small feeders into the remaining tumor blush and no change in the control right ICA runs.

Hemostasis was maintained by compression for 20 minutes and then the patient was shifted to the intensive care unit. A postembolization evaluation revealed a pulsating, localized right neck mass with no sensorimotor deficits and intact cranial nerves.

To achieve complete resection with minimal neurovascular compromise, the surgical procedure was performed by a multidisciplinary team composed of ear, nose, throat (ENT) surgeons, neurosurgeons, and vascular surgeons. The neck was explored by a 6 cm horizontal incision under general anesthesia one day after the embolization. Intraoperatively, right neck dissection and surgical resection of the right carotid body tumor were done with preservation of internal and external carotid arteries. A drain was placed in the surgical site, and the wound was closed in layers.

**Figure 3:**
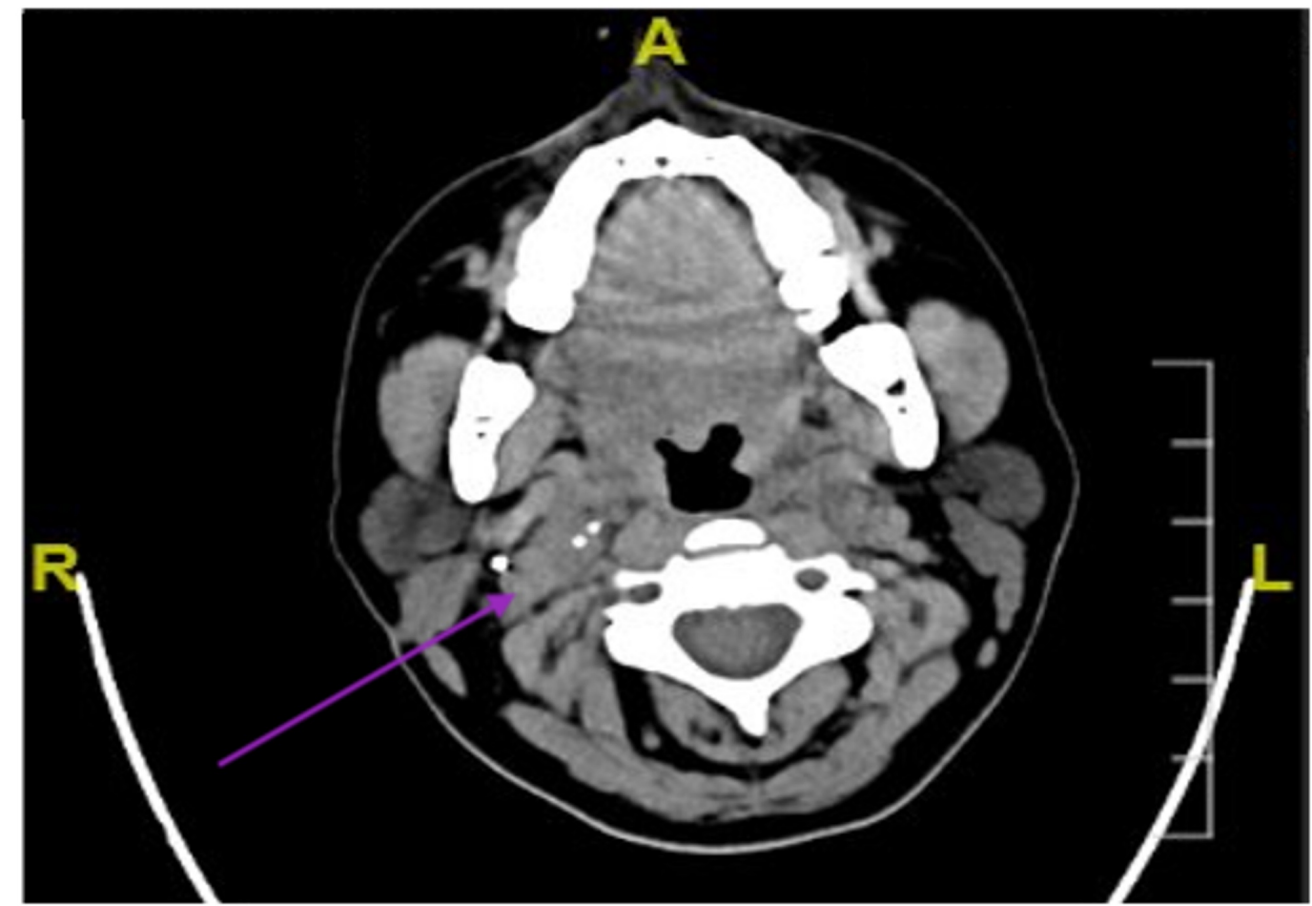
Post-surgical resection computed tomography

The postoperative course was uneventful, and her post-resection CT of the neck with contrast demonstrated non-organized fluid density seen at the surgical bed with no evidence of an enhancing residual mass in the right carotid space (Figure 3). Three days postoperatively, she was discharged with no active complaints.

## Discussion

A carotid paraganglioma is the most common head and neck tumor; over 65% of paragangliomas are CBTs. However, most CBTs are non-functional vascular neoplasms and mainly present with a slow-growing cervical mass. Therefore, duplex ultrasound is recommended as an effective diagnostic tool while a CT scan and other imaging studies are important for presurgical assessment [7].

Complete surgical resection of CBT remains the treatment of choice. However, advanced carotid body tumors are challenging to resect due to the high risk of significant bleeding intraoperatively. Consequently, that will affect the surgical outcome and the risk of neurovascular complications. Therefore, the importance of selective preoperative embolization to occlude the tumor’s feeders has been supported in a retrospective study and its surgical results were significant between the two groups, which include less amount of blood loss and shorten the operative time in the embolization group. There was no difference in the incidence of cranial nerve injury. However, embolization might lead to intense local inflammation, which necessitated reducing the surgical interval between embolization and resection [8].

Onyx is a non-adhesive liquid embolic agent, which has been used in several reported cases of paragangliomas. A direct intralesional embolization with 20 ml of Onyx was performed preoperatively in a 20-year-old woman with a carotid body tumor of Shamblin class III until devascularization was achieved. Intraoperatively, there was minimal bleeding, which facilitates complete CBT resection with the preservation of common carotid, internal, and external carotid arteries. However, the patient showed symptoms of Horner syndrome and deficits of the hypoglossal and glossopharyngeal nerves [9].

In this case report, we have performed selective pre-resection embolization using two liquid embolic agents, Onyx 18 and PHIL 25%, which achieved an embolization of 95% of the carotid body tumor supply. Postembolization, there were no neurovascular complications or evidence of cranial nerve involvement or sensorimotor deficits. Intraoperatively, complete CBT resection has been achieved with the preservation of internal and external carotid arteries.

## Conclusions

We report a case of carotid body tumor who underwent preoperative selective embolization by Onyx 18 and Phil 25% followed by complete surgical resection by a multidisciplinary team. Using these agents achieved 95% of tumor devascularization without worsening symptoms and complications. Therefore, based on this experience, we recommend the use of Onyx 18 to achieve optimal treatment for CBT. However, further studies must be conducted to prove the efficacy of Onyx in the management of carotid paraganglioma.
